# Supplementary material for: Perceived quality of care and choice of healthcare provider in informal settlements
Source: PLOS Glob Public Health. 2023 Feb 14;3(2):e0001281. doi: 10.1371/journal.pgph.0001281 (PMC10022014; doi:10.1371/journal.pgph.0001281)
Supplement: S1 Text — (DOCX) [file pgph.0001281.s002.docx]

S1 Text – Assumption that Journey are Carried Out on Foot

It is assumed that all journeys are carried out on foot when calculating the network cost. While it is obvious that not all journeys will be carried out on foot, we know that a significant majority of journeys were carried out on foot. This is concluded by the individuals’ responses to the survey questions, where they were asked to indicate the transport method used to visit their last HCP. The summarised results are shown in Table A. A sizeable minority of respondents carry out their journey using public transport. We have not modelled these instances for two reasons. Firstly, the label ‘public transport’ in informal settlements is vague as these settlements typically have many modes of transport that are publicly available (e.g., shared motorbikes, rickshaws, buses, etc.). As these modes are likely to need to be modelled differently (i.e., some run to a schedule while others do not), it is difficult to determine which mode each person took and how long they took to travel. Secondly, even if we did know the mode that each person used, we lack sufficient knowledge and data to accurately model each mode. We leave this more detailed modelling of different modes to future work.

Table A: Reported mode of transport aggregated across all four slums

| **Mode of Transport** | **Percentage of Respondents** |
| --- | --- |
| Walking | 77.2 |
| Public Transport | 18.2 |
| Private Vehicle | 1.8 |
| Bicycle | 1.5 |
| Taxi | 1.3 |
| Ambulance | <1.0 |
