# Supplementary material for: Perceived quality of care and choice of healthcare provider in informal settlements
Source: PLOS Glob Public Health. 2023 Feb 14;3(2):e0001281. doi: 10.1371/journal.pgph.0001281 (PMC10022014; doi:10.1371/journal.pgph.0001281)
Supplement: S2 Text — (DOCX) [file pgph.0001281.s003.docx]

S2 Text – Assumption that Clinics and Hospital are Comparable

A key assumption is that clinics and hospitals are comparable facility types (i.e., someone may reasonably choose between one or the other). To verify this assumption, we examine patient need, as reported by survey respondents. Table A shows the needs of patients that visit clinics and hospitals (we only list the needs that cover the top 90% for brevity and readability). We observe that the list and order of patient needs are very similar. Although there is some variance in the proportions of each need served by hospitals and clinics, this is not significant. It would be reasonable to assume that someone with any of the conditions listed would reasonably go to either a clinic or a hospital to have their needs met.

Table A: Reported needs of hospital users

| **Patient Need** | **Percentage of patients with specified need** | |
| --- | --- | --- |
|  | **Hospitals** | **Clinics** |
| Communicable disease | 32.6 | 38.3 |
| Acute condition | 22.8 | 30.4 |
| Other | 11.2 | 5.6 |
| Generalised pain | 8.5 | 5.3 |
| Chronic pain in joints | 5.1 | 3.7 |
| Maternal / perinatal | 4.2 | 5.0 |
| High blood pressure | 4.2 | 1.7 |
| Injury | 3.2 | 2.9 |
