# Supplementary material for: Perceived quality of care and choice of healthcare provider in informal settlements
Source: PLOS Glob Public Health. 2023 Feb 14;3(2):e0001281. doi: 10.1371/journal.pgph.0001281 (PMC10022014; doi:10.1371/journal.pgph.0001281)
Supplement: S3 Text — (DOCX) [file pgph.0001281.s004.docx]

S3 Text – Assumption that Journeys Originate from People’s Homes

We assume that all trips originate from the individual’s home, but this will not always be the case, as people may, for instance, visit an HCP on their way home from work. To verify whether this assumption is a fair one, we calculate the Pearson correlation coefficient between the reported travel times in the respondent survey, and the calculated travel times used as part of the access cost calculation. We aggregate the data for each city in the study, as data quality issues (e.g., non-responses, non-sensical responses) meant that sample sizes were low for each slum individually. The correlation coefficient equals 0.79 and 0.51 for Nairobi and Ibadan, respectively. Therefore, there exists a reasonably strong correlation between the reported travel times and those calculated in our study (e.g., where trips derive from people’s homes), which makes this a fair assumption.
