# Supplementary material for: Perceived quality of care and choice of healthcare provider in informal settlements
Source: PLOS Glob Public Health. 2023 Feb 14;3(2):e0001281. doi: 10.1371/journal.pgph.0001281 (PMC10022014; doi:10.1371/journal.pgph.0001281)
Supplement: S4 Text — (DOCX) [file pgph.0001281.s005.docx]

S4 Text – Defining the Access Cost

The set of dwellings within each slum are defined as the set $D$. Each $d_{i}\in D$ is a tuple containing the coordinates of an individual dwelling. The HCPs serving a slum are contained within the set $F$. Each $f_{i}\in F$ is a tuple containing each healthcare provider’s (HCP) co-ordinates and type. The individual level HCP visits are defined as the set $V$, where each $v_{i}\in V$ is a tuple containing the dwelling-HCP pair. The road network is defined as $G(N,E)$, where $E$ is the set of edges pertaining to roads in the network, and $N$ is the set of nodes pertaining to junctions. Each $e_{i}\in E$ is attributed to also include length of the road in metres.

The notion of a generalised access cost (sometimes called a generalised transport cost) is commonly used in transportation literature to measure accessibility to services in urban spaces [1] [2], and is often favoured due its simplicity of interpretation. The concept provides a unified cost to capture all the monetary and non-monetary costs associated with a trip. It is particularly appropriate for our study, which uses a data-driven methodology that aggregates the costs associated to a large set of simulated trips in each slum.

To calculate the total access cost to HCPs, we consider the following costs: the initial wait time, travel time, monetary cost associated with travel, and consultation fees. We formally state the access cost as:

$$AC=VOT\times TT+ TC+CF$$

where $VOT$ is a value of time conversion factor, to convert time to US dollars, $TT$ is the travel time, $TC$ is the cost of transportation, and $CF$ is the consultation fee. The access cost is given in US dollars.

The travel time component of the access cost is the sum of the distance to network (DTN) and the shortest path (SP) in $G$ to the HCP. DTN is defined as the straight-line distance in meters from a dwelling $d_{i}\in D$ to its nearest network access point (AP). As the road and footpath networks within slums are typically informal and multi-modal, we assume that it is possible to access the network at any point (i.e., there are no barriers or roads that are inaccessible by foot).

The shortest path in the network is obtained by using Dijkstra’s shortest path algorithm [3]. For the source node, we take the nearest node from the individual’s network access point, and for the destination node, we use the nearest node to the HCP. The algorithm will return the length of the shortest path in meters and use an estimated walking speed to convert to seconds. We therefore state:

$$TT\left( d_{i},f_{i} \right)=DTN\left( d_{i} \right)+SP(AP\left( d_{i} \right),f_{i})$$

The consultation fee ($CF$) is recorded in the individual survey data. A currency conversion factor is used to convert the fee paid from local currency into US dollars. To calculate $CF$, we sum the consultation fee charged for all visits to an HCP, which is then averaged to give the mean consultation fee per HCP. This value is then used as the $CF$ value for all access costs to that HCP. We denote the set of visits to a particular HCP as $V_{f_{i}}$. Thus, $CF$ is given by:

$${CF}_{f_{i}}=\frac{1}{\left| V_{f_{i}} \right|}\sum_{v_{j}\in V_{f_{i}}} v_{j}\left[ 'fee paid' \right]$$

# References

| 1. | Bocarejo S J, Oviedo H D. Transport accessibility and social inequities: a tool for identification of mobility needs and evaluation of transport investments. Journal of transport geography. 2012 Sep; 24: 142-54. |
| --- | --- |
| 2. | Koopmans C, Groot W, Warffemius P, Annema J, Hoogendoorn-Lanser S. Measuring generalised transport costs as an indicator of accessibility changes over time. Transport Policy. 2013 Sep; 29: 154-9. |
| 3. | Dijkstra EW. A note on two problems in connexion with graphs. Numerische mathematik. 1959 Dec; 1(1): 269-71. |
