# Supplementary material for: Perceived quality of care and choice of healthcare provider in informal settlements
Source: PLOS Glob Public Health. 2023 Feb 14;3(2):e0001281. doi: 10.1371/journal.pgph.0001281 (PMC10022014; doi:10.1371/journal.pgph.0001281)
Supplement: S5 Text — (DOCX) [file pgph.0001281.s006.docx]

S5 Text – Defining Bypass Behavior

Here we use the same definitions which have been stated at the beginning of S4 Text.

A bypass is identified when the access cost (AC) to a healthcare provider (HCP) is lower than that of the visited HCP. Formally, facility $f_{a}$ is bypassed if $AC(d_{i},f_{a})<AC(d_{i},f_{b})$ where $f_{b}$ is the visited HCP. To calculate this efficiently, we pre-calculate all the access costs for all $d_{i}\in D$ and $f_{i}\in F$. Then, to identify all the HCPs that have been bypassed during a visit, we retrieve all the access costs for the individual’s dwelling and sort in ascending order. Each HCP with a lower access cost than the visited facility is classed as a bypass. Direction is not considered, since an individual may consider HCPs in any direction.

Each bypass is scored to provide a contribution both to the bypassed and the visited HCP. This bypass contribution (BC) is the access cost to the bypassed HCP as a proportion of the access cost to the visited HCP. This means that BC indicates that bypasses are weighted differently, where bypassed HCPs that are close to the individual are given greater weights than those further away. Formally, the bypass contribution is defined as:

$$BC\left( {d_{i}, f}_{a}, f_{b} \right)=\left\{ \begin{aligned} \frac{AC(d_{i},f_{b})}{AC(d_{i},f_{a})}, &AC(d_{i},f_{a})<AC(d_{i},f_{b}) \\ 0, &AC(d_{i},f_{a})\geq AC(d_{i},f_{b}) \end{aligned} \right.$$

where $f_{a}$ is the bypassed HCP, and $f_{b}$ is the visited HCP.
