# Supplementary material for: Perceived quality of care and choice of healthcare provider in informal settlements
Source: PLOS Glob Public Health. 2023 Feb 14;3(2):e0001281. doi: 10.1371/journal.pgph.0001281 (PMC10022014; doi:10.1371/journal.pgph.0001281)
Supplement: S6 Text — (DOCX) [file pgph.0001281.s007.docx]

S6 Text – Calculating the Perceived Quality Index

We use the bypass contribution (BC) values to define an intuitive metric – the perceived quality index (PQI) – which acts as a proxy for perceived quality. The idea is that healthcare providers (HCPs) that ‘attract’ patients should be rewarded, with extra weight given to HCPs that encourage patients to bypass HCPs closer to their origin. Similarly, if an HCP is bypassed it should be penalised, with greater penalties given to those HCPs that are close to the patient’s origin. Hence, the PQI at the HCP level is defined as:

$${PQI}_{f_{a}}= \sum_{v_{i}\in V} BC\left( d_{v_{i}} , f_{v_{i}} , f_{a} \right)-\sum_{v_{i}\in V} BC\left( d_{v_{i}}, f_{a}, f_{v_{i}} \right)$$

For each bypass, the BC is subtracted from the PQI for the bypassed HCP and added to the PQI for the visited HCP. As such, the information contained within the bypasses is aggregated into the HCP level PQI, which can be positive or negative.

The intuition of our definitions for bypass behaviour and PQI is illustrated through the following example. S6 Fig shows three HCPs and two patients, who each travel to different HCPs. Patient 1 bypasses Facilities B and C to visit Facility A. Our definitions penalise these first two HCPs (B and C), with C being penalised more given its closer proximity to Patient 1. Conversely, Facility A is rewarded for ‘attracting’ Patient 1, with this reward boosted by the fact it has attracted Patient 1 despite them having nearer options available to them. Similarly, when Patient 2 visits Facility B, Facility A is penalised as it is bypassed to visit Facility B, which is rewarded for. Facility C is not penalised as it was not bypassed. The net PQI values show that Facility A is the most attractive facility, despite being bypassed once, as it has attracted Patient 1 from further away. Facility C, having attracted no patients and been bypassed once, has the lowest perceived quality.

*S6 Fig: Worked Example of the Perceived Quality Index*

Table A: Worked example of perceived quality index based on S6 Fig

| **Facility** | **Patient 1** | | | **Patient 2** | | | **Perceived Quality Index** |
| --- | --- | --- | --- | --- | --- | --- | --- |
|  | **AC** | **BC** | **Visit** | **AC** | **BC** | **Visit** |  |
| A | 10 | - | Y | 2 | 1.50 | N | (2.50 + 1.67) – 1.50 = 2.67 |
| B | 6 | 1.67 | N | 3 | - | Y | 1.50 - 1.67 = -0.13 |
| C | 4 | 2.50 | N | 6 | - | N | - 1. 0.00) = -2.50 |
