# Supplementary material for: Perceived quality of care and choice of healthcare provider in informal settlements
Source: PLOS Glob Public Health. 2023 Feb 14;3(2):e0001281. doi: 10.1371/journal.pgph.0001281 (PMC10022014; doi:10.1371/journal.pgph.0001281)
Supplement: S7 Text — (DOCX) [file pgph.0001281.s008.docx]

S7 Text – Implementation Details

We state here the values used in the implementation of our methodology.

**Value of Time Factor**

A value of time (VOT) factor is used to value an individual’s time in order to capture the inconvenience associated to accessing healthcare. This is sometimes referred to as a displacement cost and the cost the employer can be used. In our case, we will attain an average hourly wage in each site and use this to convert time to money [1].

Finding accurate data pertaining to wages for LMICs is challenging. A recent news article indicates the urban poor in Nigeria earn 2 USD per day [2]. We are unable to find any recent information pertaining to Kenya, and so we use the same value. We assume people work six hours per day.

The VOT factor is therefore given as: 0.33 USD per hour [1] [2]**.**

**Walking Speed**

Walking speed, used when calculating transitions in the network, is set at 2.5 m/s.

**Currency Conversion**

We use the following currency conversions factors, taken from Google (who source their currency conversion data from Morningstar) on 1^st^ January 2019. We use this date as the individual and HCP surveys were conducted throughout late 2018 and early 2019.

1 Kenyan Shilling = 0.0093 USD

1 Nigerian Naira = 0.0027 USD

# References

| 1. | Whittington D, Cook J. Valuing Changes in Time Use in Low- and Middle-Income Countries. Journal of Benefit-Cost Analysis. 2019; 10(S1): 52 - 72. |
| --- | --- |
| 2. | Okporua K. Independent. [Online].; 2020 [cited 2022 06 06. Available from: <https://www.independent.co.uk/arts-entertainment/photography/largest-floating-slum-lockdown-livelihoods-b745528.html>. |
