# Supplementary material for: Perceived quality of care and choice of healthcare provider in informal settlements
Source: PLOS Glob Public Health. 2023 Feb 14;3(2):e0001281. doi: 10.1371/journal.pgph.0001281 (PMC10022014; doi:10.1371/journal.pgph.0001281)
Supplement: Attachment — Submitted filename: Response to Reviewers.docx [file pgph.0001281.s009.docx]

Dear Reviewers / Editors,

We thank the reviewers for taking the time to review our work and providing actionable feedback, and particularly for the excellent references they have suggested. In the table below we address each point made in the reviews, as well as feedback from the journal. We have made appropriate revisions to the manuscript based on this feedback, and we reference where the changes have been made in relation to each point.

| **Ref number** | **Comment** | **Response from authors** |
| --- | --- | --- |
| 1 | ...it runs counter to what we experience in LMICs e.g., in Bangladesh (Ref: http://www.health-policy-systems.com/content/5/1/1; http://www.biomedcentral.com/1472-6963/11/S2/S8; HEALTH POLICY AND PLANNING LANNING;15(1):95-102 ) - **Reviewer 1** | We thank the reviewer for these interesting references, which we have read carefully. The first reference is based on direct measurement of satisfaction rather than observed health-seeking behaviour. Nor does it address the comparison between informal vs formal services which is the comparison where our findings produce consistent results. The evidence from this interesting reference therefore does not contradict our findings. The second reference is not comparable as it relates to findings from staff questionnaires. |
| 2 | ...the current findings need to be discussed in the context of findings from e.g., LMICs in Asia (relevant literature is conspicuously absent)!! - **Reviewer 1** | We have added revisions to the introduction and discussion sections in which we discuss a broader context for our work, including several studies that investigate health-seeking behaviours in LMICs and studies that investigate the association between bypassing and perceived quality. (See lines: 49, 58-69, 212 - 214, 396-401, 405, 415 - 418). |
| 3 | the ms need to be examined critically for statistical calculations of variables such as 'access cost', 'attractiveness index', 'perceived quality', 'bypass behaviour' etc. with appropriate conceptual background and appropriate references - **Reviewer 1** | We have addressed this comment in several ways. Firstly, we include a more technical description of the method in the main body of the text (some taken from the original appendices, some new to this revision) to give the reader a better technical understanding of our work (lines 87-92, 111-114, 216-226). Secondly, we have included more conceptual background around the concept of accessibility and its applicability in an LMIC setting (lines 106 - 108, 238 - 242) and we've provided more evidence to support the link between bypass behaviour and perceived quality (lines 212-214, lines 65 - 69). Finally, we have renamed what was "attractiveness index" to the "perceived quality index" to make it clearer what this term means, and provided evidence from similar studies to support our methodology in calculating this term (lines 222 - 226). |
| 4 | not clear if the QoC is better in Kemya/Nigeria than in BD (ref please)! - **Reviewer 1** | We do not claim to be calculating an objective measure of quality of care that can be compared between locations, it is rather a relative measure of perceived quality that can indicate which facilities amongst a comparable set people are most willing to incur costs (time and money) to visit within a location. Therefore, this comparison is not possible with our methods. We have added to the manuscript a disclaimer that we do not measure the technical quality of care (line 249). |
| 5 | In the limitations, quite a few critical confounder is mentioned...despite these, the argument of the paper/validity of the data should be questioned...isn't the conclusions a little too ambitious? - **Reviewer 1** | We agree that we have to make various assumptions in our work which could be regarded as confounders. We deal with the possible inferences from our conclusions in the next response below. Here we observe that our assumptions are shared with other studies in the field; for example, the assumption that travel to a facility started from home (line 446-447). We also note that we go further than other studies, and provide quantitative justification for our assumptions, through an analysis provided in the supplementary material. Finally, we point out where the assumptions are conservative, for example our finding that people are attracted to formal facilities would likely be even greater among patients with more severe symptoms. We now include evidence to support this assumption (line 433). |
| 6 | If the quality gradient suggested by the Lancet series and by our findings is confirmed, then the development of services should not be tilted in favour of smaller clinics, until quality can be better assured among such providers'. A dangerous policy proposition for the LMICs across the worls, in direct opposition to the theme of renovated PHC for UHC Call (Astana 2018)! - **Reviewer 1** | We make no pretence that our paper by itself can or should drive policy change. In fact, we explicitly say that ‘we are not arguing that international policy should turn on our single study’ (line 478). However, we think it would be a mistake not to mention possible implications of our study. First, the Astana declaration is careful not to exclude general purpose hospital clinics from the ambit of primary care. In response to the reviewer we now make this point (lines 476-477). Second, we do not rely solely on our findings but contextualise our findings with the general literature, some of it generously identified by the reviewers of our paper (see comment numbers 2 and 10). Third, our suggested policy implication aligns with one of the main recommendations from the recent Lancet Commission on service provision (McPake B, Hanson K. Managing the public–private mix to achieve universal health coverage. The lancet. 2016 Aug; 388(10044): 622-30)). Fourth, we do not make this suggestion in the results, nor do we argue that it is a corollary or conclusion. We think the purpose of the discussion is to at least consider possible implications of our findings contextualised in a broader literature. Ultimately we will be guided by the Editor on this point. |
| 7 | A key should be provided for Figure 2 - **Reviewer 2** | Thank you, we have added this. |
| 8 | Figure 3 and Figure 4 are not very clear. In fact, the quality of the figures can be improved. The units of measurement should be indicated in the figures - **Reviewer 2** | We have made improvement to all the figures except figure 1.  Figures 3, 4 and 6 have been output in a different structure (arranged in the same 2 by 2 structure as the other figures) to make them clearer and easier to interpret. Additionally figures 4 and 6 the plots are now grouped by each slum so we can draw direct comparisons and observe patterns *within* a slum, which is the more meaningful interpretation and follows how we present the results in the text.  We have updated the captions for figures 3, 4 and 6 to reflect these changes and to be more meaningful to the reader.  Some text has changed to better signpost the amended plots.  More generally we have improved the quality of the figures with consistent use of fonts and font size, updated legends, better plot alignment and to include the unit measurement where appropriate. |
| 9 | There is a lot of commentary in the presentation of the results. It is good that the authors should reduce the commentary and try to go straight forward to present the results. - **Reviewer 2** | We have reviewed the results and removed any unnecessary commentary to ensure this section is more succinct (lines 292-293, 322 - 325, 376-380). |
| 10 | The discussion section should be expanded to ensure that the evidence of this current study is compared with findings from similar studies in other low and middle income countries such https://bmchealthservres.biomedcentral.com/articles/10.1186/s12913-021-06573-3 -Bypassing primary healthcare facilities for maternal healthcare in North West Ghana: socio-economic correlates and financial implications - **Reviewer 2** | We have included the insights from this study, plus several others that investigate bypass behaviour in LMICs and health-seeking behaviour in general. These findings have been added to the introduction to give our study better context, as well as the discussion section to contextualise our findings. We thank the reviewer for drawing our attention to these important works. (See lines: 49, 58-69, 396-401, 405, 415 - 418). |
| 11 | 1. Please amend your online detailed Financial Disclosure statement. This is published with the article. It must therefore be completed in full sentences and contain the exact wording you wish to be published. a. State the initials, alongside each funding source, of each author to receive each grant. b. State what role the funders took in the study. If the funders had no role in your study, please state: “The funders had no role in study design, data collection and analysis, decision to publish, or preparation of the manuscript.” - **Journal Requirements** | We have amended our financial disclosure statement as requested. |
| 12 | Please ensure that the funders and grant numbers match between the Financial Disclosure field and the Funding Information tab in your submission form. Note that the funders must be provided in the same order in both places as well. - **Journal Requirements** | We have updated funder and grant numbers as requested. |
| 13 | Please update your online Competing Interests statement. If you have no competing interests to declare, please state: “The authors have declared that no competing interests exist.” - **Journal Requirements** | We have updated the competing interests statement as requested. |
| 14 | We do not publish any copyright or trademark symbols that usually accompany proprietary names, eg (R), (C), or TM (e.g. next to drug or reagent names). Please remove all instances of trademark/copyright symbols throughout the text, including © on page 21. - **Journal Requirements** | We have taken the symbol out. |

We thank you again for taking the time to review our paper and look forward to hearing back from you.

Sincerely,

Authors
